# Supplementary material for: Are carbon emissions trading and green financial instruments synergistic? -Comprehensive quantitative research based on content analysis
Source: PLoS One. 2024 Mar 7;19(3):e0298601. doi: 10.1371/journal.pone.0298601 (PMC10919601; doi:10.1371/journal.pone.0298601)
Supplement: S1 Data — (ZIP) [file pone.0298601.s001.zip › Data/地级市Z_F工作报告.zip!/┴╔─■/┼╠╜⌡╩╨/└┤╫╘╣1⁄2╓┌║┼í╛┬φ┐╦╩2╛▌═°í┐_2011.docx]

各位代表：

现在，我代表市人民政府，向大会报告工作，请予审议，并请市政协委员和其他列席同志提出意见。

一、2010年和“十一五”工作回顾

2010年是盘锦发展进程中极不平凡的一年，在中共盘锦市委的领导下，全市人民团结一心，奋力拼搏，战胜重重困难，实现经济社会又好又快发展，圆满完成年初确定的各项任务。

预计地区生产总值950亿元，同比增长18%；财政一般预算收入80.6亿元，增长49.7%；固定资产投资900亿元，增长65%；社会消费品零售总额 185亿元，增长18.5%；实际利用外资9.1亿美元，增长202%；外贸出口3.4亿美元，增长16.3%。主要经济指标增速走在了全省前列！

（一）项目建设取得重大进展。千万元以上项目引进213个，开工520个，竣工205个。

1．工业集群快速成长。千万吨炼油、百万吨烯烃等重大项目开始启动，世界行业领军企业台湾长春集团加盟盘锦，华锦扩建工程达产达标，盘锦正加速向全国石化 基地迈进。石油钻采设备、海工装备等大项目加快建设，装备制造即将成为新的支柱产业。“五大产业集群”拉动工业增长25.4个百分点。高新技术产品增加值 增长30%。

2．现代服务业集聚区建设成效显著。大商城市广场、鹏欣水游城等大项目加快建设，香港恒大、东方银座、广东美的等知名企业云集盘锦。湿地旅游树起崭新形 象，珠海天沐温泉城等大项目签约落地，旅游总收入增长48.5%。新开工房地产560万平方米。物流、会展、文化等产业蓬勃兴起。

3．农业现代化步伐加快。开工新建六和肉鸭加工等大项目23个，建成设施农业5万亩，新改建畜牧小区500个，新增水产小区30个，养殖大蟹15万亩。农机装备水平显著提高。战胜低温寡照、严重洪涝等自然灾害，粮食生产达到105万吨。

（二）辽滨新区建设如火如荼。全市上下同心协力发展辽滨，建设辽滨。如今，辽滨新区总体规划面积由110平方公里扩大到545平方公里，成为辽宁综合改革 试验区，石油化工、石油装备和海洋工程三大园区跻身省级产业基地。新区产业迅速壮大，知名度、影响力不断提升，宝来石化、振奥橡胶等重大产业项目发挥了巨 大的集聚效应，港口、铁路、公路、大学、医院等重大基础工程全面建设。一个充满生机与活力的滨海生态新城开始形成。

（三）城乡建设统筹推进。完成新一轮行政区划调整，城乡一体化格局加快形成。

1．产业集聚区建设迈出新步伐。规划了“2+7”经济区（园区），加大了路、水、电、气等基础设施建设的力度，吸引一大批项目进驻发展。县区经济强劲增长，实现三年倍增目标。

2．城市化进程加快。大力推进铁东老区、中央商务区等区块改造，新的城市空间正在形成。竣工中华路、泰山路等12条道路，加快中心医院、数字化城管、污水 垃圾处理等项目建设，推进绿化美化亮化，市容市貌焕然一新。田家、高升等中心镇建设步伐加快。城市化率达到65%。

3．社会事业全面进步。教育事业均衡发展，教育质量稳步提高。基本药物制度改革启动实施，公共医疗卫生体系更加健全。文化体制改革扎实推进，文化事业繁荣发展。公共服务资源加快向农村延伸，农村生产生活条件明显改善。

（四）体制机制创新实现重大突破。

——加快权力下放，理顺管理职能，大大激发了发展活力。四个县区和辽滨、辽河口两个市属经济区在新的起点上竞相发展、加快崛起。

——全面完成政府机构改革任务，优化了组织结构，明晰了权责关系；深化行政审批制度改革，盘锦成为全省审批项目最少的城市之一。

——创新“2+7”经济区（园区）体制机制。推行新的管理体制、用人机制，为大发展、快发展提供了保障。

——组建12个市直部门专业招商局，建立了以项目为主的考核机制，全市大招商、招大商的氛围更加浓厚。

（五）和谐盘锦建设扎实推进。在加快发展中，我们更加关注民生，兑现了年初承诺的46件民生实事。

1．投入3.2亿元，办理了12件就业和社会保障实事。实名制就业5.3万人，转移农村劳动力2.2万人，零就业家庭动态为零。新型农村养老保险启动实施，农村低保等4类标准提标工作全面完成，农村敬老院、儿童福利院等加快建设。

2．投入7.8亿元，解决了上学难、看病难、住房难等14个问题。资助贫困家庭学生1.7万名，新农合报销限额由3万元提高到4万元，兴建廉租房13.5万平方米，改建农村土危房600户，弱势群体大病救助等问题得到有效解决。

3．投入4.6亿元，落实了20项环境改善工程。植树造林4.5万亩。改造小街小巷71条，更新公交车辆110台。新建一批农家店、农资店和文体活动中心等服务设施，农村饮水安全问题得到解决，有线电视“屯屯通”在全省率先实现。

（六）民主法制建设不断加强。坚持依法行政，自觉接受人大法律监督、工作监督和政协民主监督，认真听取各民主党派、工商联、无党派人士的意见和建议，密切 与工会、共青团、妇联等人民团体的联系，推进决策科学化和民主化。办理人大代表建议88件、政协提案128件。加大政务公开、行政监察和审计监督力度，勤 政廉政建设进一步加强。全力维护社会稳定，公共安全秩序得到强化和发展。法制教育广泛宣传。重特大事故和伤亡人数明显下降。信访考核连续6年全省第一。突 发公共事件应急能力显著增强。国防动员、双拥优抚工作取得新成绩，驻盘部队和广大民兵预备役部队作出了新贡献。统计工作经验在全国推广，人口与计划生育、 民族宗教、外事侨务、人防、物价、质监、贸促、档案、地方志、住房公积金等工作不断加强，妇女儿童、老龄、残疾人等事业实现新的发展。

各位代表！2010年的工作为“十一五”画上了圆满的句号。过去的五年，是团结奋进的五年，是克难攻坚的五年，是历史巨变的五年。

经过五年的奋斗，经济转型进入了新阶段。

——综合实力快速攀升。地区生产总值自2008年开始两位数增长，经济总量是“十五”期末的2.1倍。固定资产投资、财政收入等主要经济指标都实现了高速增长。我们已经昂首跨入加快发展的快车道！

——发展活力显著增强。以改革促开放、促发展、促转型的格局基本形成，利用外资增幅连续三年走在全省前列。成功举办国际湿地旅游周、石油装备制造与稠油技术博览会等重大活动，盘锦树起开放发展的新形象。

——经济结构明显优化。三次产业比重调整为8.3：68：23.7。接续产业迅猛发展。节能减排实现控制目标。民营经济撑起半壁江山，成为推动发展的生力军。

经过五年的奋斗，盘锦开启了向海发展的新征程。

——大开放格局基本形成。辽滨沿海经济区从荒滩上拔地而起，盘锦迎来了崭新的海洋时代，成为辽宁对外开放的热点，成为辽宁大振兴的焦点。

——大城市框架迅速拉开。从南到北，辽滨水城、大洼温泉城、兴隆台新城、双台子新城、盘山新城五个生态新城展现了新的风貌，滨海大城市建设迈出了坚实步伐。

——基础设施建设取得突破性进展。凝结着几代人梦想的盘锦海港开港通航，沿海亿吨大港正成为现实。营盘客专、疏港铁路、沈盘铁路等加快建设，辽河大桥建成通车，向海大道全线贯通，盘锦正加速向枢纽城市迈进！

经过五年的奋斗，社会建设呈现出和谐奋进的新局面。

——人民群众生活水平持续攀升。城乡居民收入分别达到21035元、9700元，是2005年的1.9倍。城乡人均消费支出分别达到13923元和5014元，人均储蓄存款余额达到3.96万元，居全省前列。

——公共服务和社会管理显著加强。累计投入财政资金140.4亿元，占总支出比重达到44.6%。就业、居住、养老、医疗等民生问题得到有效解决，科教文 卫和社会管理等工作显著进步，盘锦先后获得全国社会治安综合治理优秀市、全国双拥模范城、全国创建文明城市工作先进市等荣誉称号。

全市上下始终激情满怀，奋发有为。全市人民期盼发展、追求美好生活的强烈愿望，正加快成为建设滨海新盘锦的生动实践！

各位代表！回顾过去的五年，盘锦的变化有目共睹，盘锦的发展振奋人心！在此，我谨代表市政府，向全市各族人民，向人大代表、政协委员，向各民主党派、人民 团体和各界人士，向离退休老领导、老同志，向人民解放军、武警驻盘部队官兵、公安干警，向辽河油田、长城钻探、辽河石化、华锦集团等驻盘单位，向关心、支 持和参与盘锦建设发展的海内外朋友，致以崇高的敬意和衷心的感谢！

各位代表！我们也清醒地认识到，盘锦经济社会发展还面临许多困难和问题。经济结构不尽合理，转变发展方式任重道远；改革开放尚需加大力度，自主创新能力不 强，经济增长的动力和活力有待提升；民生保障与人民群众的期待还有较大距离；政府自身建设有待加强，思想需更加解放，工作需更加务实。对此，我们必须认真 加以解决。

二、“十二五”时期的展望

《盘锦市国民经济和社会发展第十二个五年规划纲要（草案）》已经制定，请各位代表予以审议，请政协委员提出意见。基本思路是：全面贯彻落实科学发展观，按 照市委五届十六次全会部署，以向海发展、全面转型为主线，以改革开放为动力，着力推进沿海开发开放、城乡一体化、和谐盘锦建设，实现科学发展、创新发展、 和谐发展、跨越发展。

各位代表！未来的五年，是盘锦跨越追赶极为重要的历史时期，机遇在前，使命在肩。面对“十二五”发展的宏伟蓝图，面对全市人民的期待与重托，我们要始终保 持锐意进取、锲而不舍的干劲和拼劲，一刻也不放松，一时也不停留，团结和带领全市人民，以更加饱满的激情投入新的奋斗！

“十二五”期间，我们就是要：立足新起点，谋求新发展，实现新跨越。

立足新起点，就是树立更高理想、瞄准更高目标，坚持：

——向海发展不动摇；

——抓重大项目不动摇；

——生态城市建设不动摇；

——改善民生不动摇；

——体制机制创新不动摇。

谋求新发展，就是按照市委、市政府“建设滨海新盘锦”的战略部署，搭建三大发展空间，实现重点突破，带动全局。

——南部，把545平方公里辽滨沿海经济区作为战略支点，建设“一城、一区、一园”。

一城，即：130平方公里辽滨水城；

一区，即：315平方公里产业区，包括石油化工、石油装备、海洋工程、高新技术四个产业园和大洼临港工业区；

一园，即：100平方公里大型生态湿地公园。

——中部，以向海大道为依托，重点建设“五园、四城”。

五园，即：25平方公里辽宁北方新材料产业园、25平方公里盘锦精细化工（塑料）产业园、35平方公里石油装备制造产业园、20平方公里石油化工产业园和20平方公里盘锦（高升）现代农业开发区；

四城，即：50平方公里盘山生态新城、40平方公里双台子辽河生态新城、50平方公里兴隆台生态新城和68平方公里大洼温泉城。

——西部，以世界最大的滨海芦苇湿地为依托，建设968平方公里辽河口生态经济区和239平方公里红海滩湿地旅游度假区。

在三大空间的发展上，我们要紧紧把握五个重点：

1.我们要用“世界的高度、未来的眼光、现代化的标准”，科学编制各项规划。

2.我们要建立充满活力、富有效率、更加开放的体制机制，成为东北最具吸引力的地区之一。

3.我们要建设竞争优势明显的产业集群，成为东北最具影响力的新型产业基地之一。

4.我们要保护好、利用好、发展好生态湿地资源，建设生态盘锦、绿色盘锦。

5.我们要全力推进城市化，建设滨海大城市，形成共建共享和谐社会的生动局面。

实现新跨越，到2015年：

——主要经济指标增速走在全省前列，力争综合实力进入辽宁第一方阵。

——产业结构更趋合理，现代产业体系初步建立，质量、效益、速度同步提升，成为全国资源型城市可持续发展示范区。

——“五个城市组团”初步建成，四通八达的大交通体系基本确立，城市化率达到80%，成为环境优美、充满活力的滨海生态城市。

——公共服务和社会管理进入全国先进行列，城乡居民人均收入保持全省前列，人民生活水平一年更比一年好！

各位代表!我们正置身科学发展的伟大时代，肩负跨越发展的神圣使命。只要我们认准目标，脚踏实地，锐意进取，就一定能够实现再造一个新盘锦的光荣与梦想!

三、2011年主要工作任务

今年是实施“十二五”规划的第一年，也是实现盘锦跨越发展的关键一年。我们必须抓住重大问题、关键问题，全力实施五大战略：

1．重大项目推动战略。就是要全力建设一批具有“发动机”效应的重大项目，用大项目推动大发展、快发展。

2．现代服务业拉动战略。就是要做大做强旅游、商贸、物流、文化产业，聚集人气，拉动城市发展。

3．辽滨新区突破战略。就是新区要用不达目的不罢休的干劲和拼劲，在利用外资、重大项目和基础设施建设上实现重大突破。

4．三大区域并进战略。就是各个县区、经济区、重点园区一起发力，大干快上，竞相发展。

5．体制机制创新战略。就是用新的体制机制，使人人有压力、人人有动力，始终让盘锦充满活力。

全市经济和社会发展主要预期目标是：地区生产总值增长17%；固定资产投资增长30%；财政一般预算收入增长18%；社会消费品零售总额增长18%；外贸 出口增长12%；城市居民人均可支配收入增长13%；农民人均纯收入增长16%。我们要力争主要经济指标增速走在全省前列。

重点抓好七项工作：

（一）全力推进辽滨新区开发开放。辽滨新区是盘锦对外开放的龙头。要以高标准的规划引领科学发展，以前所未有的魄力推进建设。

1．基础设施建设要有新突破。重点抓好“四项工程”：

——港口工程：港口是新区建设的“牛鼻子”工程，是盘锦走向世界的关键。抓紧建设7个5万吨级码头，推进25万吨级原油码头及航道工程；确保完成一类口岸晋升，大幅提高港口通关效率，实现港口和口岸功能大提升。

——交通工程：建成疏港铁路，加快建设沈盘铁路，开工建设中华路至辽滨、田庄台至二界沟等快速干道，完善新区内部路网，实现交通环境大提升。

——公共服务工程：建成省实验学校辽滨分校、盛京医院辽滨分院，加快建设盘锦大学、体育中心等重大项目，加强供暖、供气、供电等功能配套，实现公共服务环境大提升。

——生态工程：高标准完成百万平方米景观绿化和水系治理，抓紧荣兴水库、三角洲水库水源工程与景观建设，实现生态环境大提升。

2．重大项目建设要有新突破。新上2亿元以上项目50个、10亿元以上项目9个，引进世界500强或行业世界100强企业5家以上，实际利用外资6亿美元以上。

——重点瞄准香港、台湾和东南沿海等地区，着力引进一批具有“发动机”效应的重大项目。

——创造一切条件，推进千万吨炼油、百万吨烯烃、深水海工装备等重大项目，树起石油化工、石油装备、海洋工程三大省级产业基地新形象。

——启动大型商业设施、住宅小区等一批大项目，加快人口聚集。

3．体制机制创新要有新突破。用足用好省综合改革试验区各项政策，赋予新区市级经济管理权限，建设最具效率、最有活力的开发区。

——实行大部制管理模式，一局多能、一人多职、一职多责，提高办事效率。

——实行全新的选人用人机制，公开竞聘，绩效管理，能者高薪，打破“铁饭碗”，打破“大锅饭”。

——成立综合服务中心，提供从签约、审批到建设、投产的全方位、全流程、全天候服务。

今年是新区建设特别关键的一年，要保证各项主要经济指标实现翻番，全面完成任务。再经过四年努力，基本建成一个新的滨海生态新城，成为国家级开发区，成为科学发展、快速发展的排头兵。

（二）集中一切力量抓项目。项目就是发展、就是税源、就是民生，必须坚定不移地抓项目，特别是重大项目。

1．狠抓招商引资。对外开放的重要任务就是招商引资，这决定着盘锦的未来，决定着各项指标的完成。全市上下都要把更多的时间、更多的精力放在招商引资上， 大规模地“走出去”、“请进来”，着力引进一批有实力、有影响力的大项目、好项目，以大招商推动大开放、大发展。力争实际利用外资15亿美元，引进内资 600亿元。

2．狠抓项目开工。把2011年作为项目的“开工年”，必保千万元以上项目开工1000个，竣工260个。

——全力推进台湾长春石化、振奥丁基橡胶、台湾见龙EPS等石油化工大项目开工建设。

——全力推进“气化辽宁”工程、石油总部基地、海上风机安装船等装备制造大项目开工建设。

——全力推进海兴塑料、金田塑料、北方戴纳索丁苯橡胶等新材料大项目开工建设。

——全力推进香港东方银座、香港利港、香港荣盛、广东美的、浙江银亿等商贸地产大项目开工建设。

对每个项目，都要建立责任制，明确一名市长、一名县区长、一名局长，一包到底，让项目在最短的时间内开工、竣工。

3．狠抓土地和资金保障。

——全力做好土地供应。加大力度填海造地，吹填规模达到100平方公里。全力争取建设用地指标，加快推进土地增减挂钩，坚决收回闲置土地，节约集约用地，力争成为全省科学用地的先进典型。

——全力做好融资服务。引导金融机构扩大信贷投放，推动信贷持续增长。抓好基金对接，实现基金引进的重大突破。搞好城建债券发行，科学运用BT、BOT等融资手段。发展村镇银行、小额贷款公司和担保公司，尽快让市商业银行成为区域性大银行。

4．狠抓项目服务。牢固树立“服务就是生产力、服务就是竞争力”的理念，真心尊重企业、服务企业，建立以企业满意度为标准的考核监督机制，实行全程服务、 保姆式服务，做到服务“零障碍”。我们就是要说到做到，讲诚信，讲信誉，无论对外还是对内，无论是新企业还是老企业，都同样对待，以信誉赢得信任，以服务 赢得发展，让盘锦成为项目集聚的洼地、投资的热土。

（三）加快工业大振兴。工业是推动发展的主力军。狠抓“工业五项工程”，做大做强工业经济。

1．园区建设要上新台阶。一是全力推进基础设施建设，所有园区都要迅速具备招商条件。二是推动项目加快向园区集中，培育特色产业集群。三是加快体制机制创 新，实行封闭管理，使园区有压力、有动力、有创造力。每个重点园区都要引进投资超10亿元项目2个以上，其中3个省级园区都要引进世界500强或行业世界 100强企业1家以上。

2．企业集团培育要上新台阶。

——全力支持辽河油田、长城钻探、辽河石化、华锦集团、宝来石化等企业上项目、扩规模，加快培育主营业务收入超百亿、超500亿元的大企业大集团。

——全力支持辽河油田多种经营企业，振兴、和运等现有老企业对外合资合作，全力促成陕西重汽、济南重汽等重大合作项目签约落地，做大做强一批骨干企业。

——全力推进北京联东、长沙巨星等工业地产大项目，开发工业地产200万平方米，为中小企业发展提供空间，壮大非公有制经济。

——全力支持企业上市融资，必保上市企业2家以上。

3．科技创新要上新台阶。

——全力支持辽河石化稠油加工研究院、和运合成橡胶研究院、辽宁陆海石油装备研究院等科研机构建设，打造一批具有国际一流水平的研发中心。

——大力推进产学研合作创新，加快高新技术产业化和产业技术进步，高新技术产品增加值增长30%。

——大力推进企业并购、技术引进，加快科技企业孵化器建设，培育一批高新技术企业。

——以科技创新引领新兴产业发展，着力抓好新材料、光伏产业、新能源汽车等重点项目。

4．节能减排要上新台阶。牢固树立“宁可牺牲GDP也要保护环境”的意识，狠抓行业节能降耗、先进技术应用、项目环评管理，加快淘汰落后生产能力，坚决实现节能减排目标，发展循环经济、低碳经济。

（四）推进现代服务业大发展、快发展。把发展服务业作为刻不容缓的重大任务，狠下功夫，紧抓不放。

1．大力发展旅游业。建设国内外知名的旅游城市和湿地休闲之都，是盘锦的优势所在。

——旅游度假区建设要有大进展。加快建设红海滩湿地旅游度假区和辽河口生态旅游集聚区，全力抓好志高动漫主题公园和海洋主题公园、天沐温泉城等重大项目，用大规划引领大建设，用大项目、大开放推动旅游业大发展、快发展。

——旅游产品培育要有大进步。加快旅游资源整合与开发，策划辽河水上游、休闲游、农家乐等一批旅游精品，进一步叫响“湿地之都”品牌。办好第四届国际湿地旅游周，精心组织湿地观鸟月、湿地风光摄影大赛等活动。

——城市服务形象要有大提升。加大力度引进和建设五星级酒店，显著提升接待能力和服务水平。加大旅游宣传推介力度，让全中国、全世界都了解盘锦、青睐盘锦。

2．大力发展商贸服务业。加快商业基础设施建设，全力推进深圳茂业、香港佳兆业、红星美凯龙、兴隆商业综合体等品牌商贸企业和项目落户盘锦，构建中央商务区、辽河新城等特色商区，打造“1小时经济圈”商贸中心。

3．大力发展现代物流业。发挥区位、交通和产业优势，以港口为牵动，大力推进港口物流园、石化物流园等载体建设，鼓励物流服务从生产加工企业中分离，抓紧推进新加坡恒阳石化物流、益海嘉里粮食物流等重大项目，加快引进南京雨润等大型物流企业，打造区域性物流中心。

4.大力发展文化产业。依托辽河文化、湿地文化、石油文化和生态农业文化，全力推进田汉剧场、酷贝拉少儿体验中心、华强影视基地、森禾花卉公园等重大项目，建设辽河文化产业园、田庄台古镇等产业基地，尽早把文化产业培育成为战略性支柱产业。

5．大力发展职业教育。实施人才工程，加大人才培养和引进的力度，迅速启动20平方公里大洼职教城，建设全国一流的职业教育基地，为盘锦大发展、快发展提供人才保障。

（五）加快发展现代农业。农业是保发展、稳民心的基础产业，必须加大投入，促进农业增效、农民增收。

1．加快农业对外开放。用工业的理念谋划农业发展，启动建设盘锦（高升）现代农业开发区，多渠道吸引国内外资本投向农业，推进农业规模化、标准化和市场化。着力引进一批农业产业化龙头项目，两县要各上投资超10亿元的重大项目1个。

2．加快发展生态高效农业。大力实施百万亩优质水稻和百万亩河蟹基地工程，突出抓好精品培育和品牌管理，创建优质水稻高产示范区40个，养殖大蟹30万 亩，使之成为带动农民致富的龙头。启动新一轮“菜篮子”工程，新增设施农业2万亩，新建改建畜牧小区500个。强化农产品质量安全管理，做好动植物疫病防 治。

3．加快提升农业装备与管理水平。用现代物质条件装备农业，加强农业基础设施建设，推进水稻生产和芦苇收割全程机械化，提高农业综合开发和防灾减灾能力。用现代经营形式推进农业，发展农民专业合作社，培育各种社会化服务组织，建立健全土地承包经营权流转市场。

（六）加快城乡一体化进程。高标准编制城乡一体化规划、城市总体规划和专项规划。

1．大力推进城市和城镇建设。

——加大新城建设力度。大力引进大国企、大民企、大外企，加快生态新城建设和老城区改造，壮大和提升城市规模与形象。全面开工160万平方米回迁楼，尽快让老百姓住上新居。

——加大新市镇建设力度。遵循重点突破、典型引导的原则，率先抓好高升、田家等6镇试点建设，加快发展田庄台、赵圈河和欢喜岭三个特色旅游城镇。妥善解决动迁农民就业、住房、供暖、社保等问题；深化户籍制度改革，引导农民加快向城市、中心镇、农民新区聚集。

2．大力推进基础设施建设。

——加快建设营盘客专和阜盘高速，改造高速公路大洼出口，启动盘锦火车站改造工程，着力推进西外环、庄林线等15项公路工程，打造区域交通枢纽。

——高标准改造建设金城路、双绕街等18条市区街路，突出抓好林丰路及跨河大桥，畅通市区路网。加大力度改造建设地上、地下城市配套基础设施。

——加快华润热电、市中心医院、城乡电网改造、大伙房输水等工程进度。尽早建成覆盖城乡的污水、垃圾处理系统。开工建设石油博物馆、休闲广场等公共服务项目。

——抓好辽河、一统河等5条水系治理和景观建设，重点规划建设12公里辽河城区段，使之成为生态景观公园，成为百姓休闲的好去处。改造湖滨公园，打造东北最具特色的平原公园。开展绿色村庄、绿色社区、绿色企业、绿色校园和绿色通道等活动，绿化造林4.5万亩。

3．大力加强城市管理。实施城乡网格化管理，推进数字化城管，构建大城管格局。严格综合执法，狠抓市容卫生、交通秩序等专项整治，合理布局便民服务设施，使城乡美化净化绿化达到新水平。全力推进社会和城市管理创新，城市管理与公共服务要上台阶、上水平。

城乡一体化，事关发展全局，事关人民福祉。我们要把城乡一体化作为重大民生工程，带着感情，带着责任，下更大的决心、花更大的气力，让老百姓过上更好的生活。

（七）倾情倾力改善民生。时刻把群众安危冷暖装在心里，办好10项民生工程。

1．就业工程。免费就业培训5万人次，新增城镇就业4.2万人，转移农村劳动力2万人。扶持自主创业2000人。继续做好复转军人安置和随军家属就业工作。

2．社保工程。扩大新型农村养老保险覆盖范围。做好企业退休人员基本养老金、在乡复员军人优抚补助等提标工作。启动城镇居民门诊医疗保险统筹。妥善解决失地农民社会保障问题。

3．扶困工程。城市居民低保月人均提高到345元。决不让贫困家庭学生因贫失学，从学前教育到高等教育，从学费到生活费，全程资助。加大精神病人、残疾人等弱势群体的救助力度。

4．安居工程。建设公租房200套，改造农村土危房400户。支持油田边远散矿区职工迁转。抓好百万平方米供热计量改造。新建改建吴家、谷家、高家等排水泵站，着力解决市区内涝问题。

5．教育工程。着力提高教学质量，推进教育事业均衡发展。加强校园周边管理。解决农村中小学校车问题。重视学前教育，新建标准化幼儿园15所。开工建设辽河油田第一高中教学楼。

6．医疗工程。实行新农合“一卡通”即时结报制度，报销最高限额提高到10.5万元。加强疾病预防控制。对农村孕妇住院分娩予以补助，抓好贫困白内障患者免费救治、农村适龄妇女免费体检等惠民项目。

7．文体工程。建设12家乡镇综合文化站，建立农家书屋图书流通机制。完成3万户电视数字化整转工作。建成全民健身活动中心。改造提升盘锦体育场，办好第六届全民运动会。

8．便民工程。加大小街小巷改造力度，消灭城区摸黑路。推进城乡客运公交化，拓展3条城乡公交线路，加快更新公交车辆。全面解决农村公路养护与维修问题。实现房屋产权办理一体化。完善市公共行政服务中心窗口功能，提高服务企业和群众的能力。

9．养老工程。推进养老社会化，新增养老床位400张。农村五保户集中供养年人均标准提高到4368元，分散供养标准提高到2912元。

10．平安工程。妥善解决信访热点难点问题，依法有效化解社会矛盾，健全社会治安防控体系，争创全国综合治理优秀市。加强法律援助和司法调解。千方百计地做好物价稳定工作。切实抓好生产安全、食品药品安全、消防安全和交通安全。

我们就是要从群众最关心、最迫切的问题入手，竭尽全力为老百姓办实事、办好事、办真事，让群众得到更多实惠！

各位代表！实现“十二五”的奋斗目标，完成今年的工作任务，政府责任重大，使命光荣。

我们必须牢牢树立发展意识。始终满怀激情，抓住前所未有的机遇，开创前所未有的事业。不断解放思想，大胆地试、大胆地闯、大胆地干，用创新和发展解决前进中的问题。

我们必须牢牢树立责任意识。敢于担当、敢于决策，走进矛盾、破解难题，真正把决策部署变成工作成效。多想事、多干事、干成事。

我们必须牢牢树立效率意识。转变作风，求真务实，始终突出一个“快”字，定了就办、办就办好。狠抓绩效管理，实施全过程的动态考核，以结果论英雄，以业绩论英雄。

我们必须牢牢树立法制意识。自觉接受人大法律监督、工作监督和政协民主监督，广泛听取方方面面的意见和建议，不断提高政府科学理政、民主施政、依法行政的水平。

我们必须牢牢树立清廉意识。全力推进行政权力公开，让权力在阳光下运行。坚持用制度管人、管事，做到既干事又干净，坚决反对铺张浪费，努力建设廉政勤政的服务型政府，让人民信赖，让人民满意！

各位代表！盘锦正站在新的历史起点上，朝着加速崛起的目标迈进。我们坚信，有市委的坚强领导，有勤劳智慧的130万盘锦人民的共同奋斗，2011年我们一定能够迈上一个新台阶，“十二五”的宏伟蓝图一定能够如期实现，全市人民生活一定会更加幸福美好！
